# Clinical intuition in psychology through the prism of personalized psychiatry

**DOI:** 10.3389/fpsyg.2023.1111250

**Published:** 2023-04-03

**Authors:** Christophe Gauld, Yassmine Masri, Pierre Fourneret

**Affiliations:** ^1^Service de Psychiatrie de l'Enfant et de l'Adolescent, Université de Lyon 1, Lyon, France; ^2^Institut des Sciences Cognitives Marc Jeannerod, UMR 5229 CNRS & Université Claude Bernard Lyon 1, Lyon, France

**Keywords:** clinical setting, psychology, precision medicine, personalized psychiatry, modeling

## 1. Introduction

The concept of personalization in medicine dates back to the 1940s, when some theorists' attempts to find alternatives to the biomedical paradigm were already underway. Based on these early developments, the foundations of the biomedical paradigm can be traced back to personalism, developed by Mounier in the 1930s, to Balint (1969) person-centered approach, or to Mezzich et al. (2011) person-centered integrated diagnosis. Personalized medicine was initially presented as a model of comprehensive, holistic and systemic medicine (Topol, 2014; Lemoine, 2017a), whilst the current term of personalized medicine first appeared in 1999 as part of a genetic consortium. The popularity of the concept of personalized medicine in clinical practice, health policies and public discourse is therefore a relatively recent phenomenon—largely stimulated by advances in genomics and molecular biology (Lemoine, 2017a,b).

Whilst these different approaches give various definitions of personalized medicine, it is classically defined as treating “the right patient with the right treatment at the right time”. This definition is reflected in the “5R rule”: the right patient, the right medicine, at the right dose, in the right way, at the right time (Giroux, 2017). A consensual definition of the National Research Council defines personalized medicine as “the adaptation of medical treatment to the individual characteristics of each patient” [National Research Council (US) Committee on a Framework for Developing a New Taxonomy of Disease, 2011]. Finally, other proposals suggest that personalized medicine could be considered as a kind of “4P medicine”: personalized, preventive, predictive, participatory (Hood and Flores, 2012; Jakovljevic and Jakovljevic, 2019).

In psychiatry, various paradigms aligned with the principles of personalized medicine can be identified. These approaches include the emergence of new nosologies (e.g., Research Domain Criteria or Hierarchical Taxonomy of Psychopathology), and the structuring of research around biomarkers, endophenotypes, molecular signatures, as well as neuroscience and computational psychiatry (Cuthbert and Insel, 2013; Kotov et al., 2017). From these recent developments, numerous critiques of personalized psychiatry have emerged, e.g., as a regime of (exaggerated) promises, redistribution of financial support, reductionism around big data and biomarkers, potential lack of solidarity in access to health care and patient empowerment, imbalance between the (precise) individual and (potentially neglected) populational levels. In addition, questions have been raised about the benefit of individualized treatment (“How far?”) and its triviality (“Is not all care already personalized?”)—this last point constituting a key axis of the following developments.

Based on the potential contributions of personalized psychiatry and considering these criticisms, in this Opinion article intended to encourage constructive discussions on these cross-topics, we aim to explore the similarities between the fuzzy concept of clinical intuition (as an embodied clinical model) and the model offered by the framework of personalized psychiatry. Specifically, we will show that the embodied model carried out by the clinician in his/her daily practice does not substantially differ from the model proposed by the apparently innovative personalized psychiatry. We aim to show that this demonstration has profound implications for both understanding of personalized psychiatry and clinical practice in psychology. These involvements allow research and clinical practice to mutually join in a translational perspective centered around clinical intuition and personalized psychiatry.

## 2. Triviality of the personalization as the core of care

Within medicine, and even more in clinical psychology, the interest in personalization has a long history, and continues to constitute an integral part of the psychological and psychiatric disciplines today. How could one imagine the establishment of a therapeutic relationship without natural personalization? Can we imagine a caregiver who does not essentially offer personalization in terms of diagnosis and care? Is the clinical intuition of the caregiver not *always* dedicated to the personalization of care?

These kinds of questions are whether the addition of the term “personalized” to the term of “medicine” (or “psychiatry” or even “psychology”)—with all the conceptual commitments of this association—could in reality be no more than a pleonasm (triviality), or form a tautology (reinforcement)? A pleonasm implies that using these two terms would be redundant (i.e., it is using more words as necessary), while a tautology consists of associating two terms (i.e., medicine and personalization) to mutually reinforce their common sense (i.e., say similar thing twice). In this Opinion article, we will sustain both the two possibilities: the term (and its concepts) of personalized psychiatry should be certainly considered as a pleonasm; however, it is also a tautology if we consider it as a model for clinical intuition. We will develop below the relations between personalized psychiatry and psychological clinical intuition.

## 3. Modeling and clinical intuition

Psychiatry is historically and intrinsically personalized (Gurwitz and Weizman, 2004; Ozomaro et al., 2013). However, the paradigm of personalized psychiatry has differences with psychiatry. What are these differences? Our answer is based on understanding of *clinical intuition* (or “clinical internal model”), allowing caregivers to naturally personalize care (Kim and Ahn, 2002; Aboraya, 2007; Bhugra et al., 2011; Demazeux, 2019). Clinical intuition can be defined as the embodied model of a clinician (i.e., as an internal psychological, theoretical or conceptual model), belonging to the clinician, and allowing him/her to act in clinical practice (Minsky, 1965). Clinical intuition is a fuzzy concept, which groups together a set of variables, parameters, theories, backgrounds, chances, serendipities, as well as contingencies and laws (Cartwright, 1999; Sadegh-Zadeh, 2000; Kendler, 2005).

At least three perspectives have sought to identify clinical intuition. The first comes from clinical psychology, and in particular from a set of criteria proposed by Professor Paul Everett Meehl, a well-known clinical psychologist. According to Meehl, clinicians would perform certain unique and unduplicable functions that literally have no competitor. The art of clinical decision-making would be absolutely specific to clinicians because of the existence of the following six factors: open-endedness (i.e., clinical predictions are based on open questions), unanalyzed stimulus-equivalences (i.e., clinical predictions are based on perceptual Gestalts), empty cells (i.e., clinical predictions are possible even in the absence of explicit relevant factors), theory mediation (i.e., clinical predictions are based on hypothetical and non-formalizable mental constructs), insufficient time (i.e., clinical predictions must be reached within a very short time) and highly configured functions (i.e., clinical predictions are subject to extremely fine discrimination) (Meehl, 1959, 1967). These unduplicable functions performed by clinicians show some of the specificities of clinical intuition.

The second perspective seeking to identify clinical intuition comes from medical pedagogy. It refers to the different steps necessary for the development of such a clinical intuition, understood as a clinician's embodied model. These steps have been rigorously distinguished into four steps: (i) clinicians collect clinical variables (e.g., feature selection, labeling or relationships between variables), (ii) according to their individual theoretical background (e.g., Evidence-Based Practice or personality of the clinician), (iii) continually training their internal model (e.g., through theoretical or encountered clinical cases), (iv) this internal model being itself subject to uncontrollable factors (e.g., cost, time, or institutional pressures) (Spitzer, 1983; Bhugra, 2008; Bhugra et al., 2010; Martin et al., 2022).

The third perspective seeking to better understand clinical intuition comes from cognitive psychology, and in particular from the literature on cognitive biases specific to the clinical intuition of psychiatrists. A huge immense literature is interested in the bias of clinical intuition and reasoning, shedding the light on its functioning, with five main types of biases: availability biases (i.e., a tendency to give priority to clinical events that are easily accessible in memory, because they are frequent), biases confirmation (i.e., a tendency to favor clinical information that confirms the clinicians' prior beliefs), anchoring biases (i.e., a tendency to rely on initial clinical information to assess subsequent information), projection (i.e., a tendency to assume that patients share the same motivations and perspectives as clinicians) or a halo effect (i.e., a tendency to assess a patient based on initial impressions or a single aspect) (Blumenthal-Barby and Krieger, 2014; Ehrlinger et al., 2016; O'Sullivan and Schofield, 2018; Ozdemir and Finkelstein, 2018; Acciarini et al., 2020).

These three perspectives seeking to define clinical intuition underline the difficulty of giving it a precise definition. Nevertheless, this difficulty in explaining and defining clinical intuition, which ranges from clinical meaning to gestalt recognition, seems necessary for the clinician to be able to verbalize his/her choices and decisions, for pedagogy in psychology and medicine, as well as for research in psychology.

We propose that the intimate specificity of the current term of personalized psychiatry could offer an operational modeling of this clinical intuition. In other words, the core of personalized psychiatry would correspond to the vaguely conceptualized notion of clinical intuition. Personalized psychiatry could thus allow to refine clinical intuition.

Subsequently, we will explain clinical intuition modeling. What are the elements to be modeled? Should we integrate environmental, social, or cultural factors with physiological and semiological factors? Should we integrate clinician and patient subjective factors, in order to pluralistically consider both individual, mechanistic and environmental elements? To answer these questions, we propose a framework for understanding clinical intuition around the issues of precision and stratification.

## 4. Discussion

### 4.1. Personalization is modeling

Within current research practice, the question of whether personalized psychiatry should integrate both individual, mechanistic and environmental factors can be split according to two methods. Both of these methods constitute sub-parts of personalized psychiatry (Fernandes et al., 2017; Arns et al., 2021; Zanardi et al., 2021; Passos et al., 2022): precision and stratification. We will see that the two methods from personalized psychiatry can strongly support and refine the understanding of clinical intuition.

Thus, first, some research communities in personalized psychiatry tend to embrace a logic of precision, which aims to identify biomarkers, and especially physiological factors (Fernandes et al., 2017). In parallel, other research communities tend to adhere to a stratified psychiatry, which aims to create the most refined and homogeneous subgroups of patients (Arns et al., 2021). This method is based on the logic of stratification.

These methods are intimately complementary: stratification requires identifying biomarkers (i.e., relying on precision) to differentiate its subgroups, and precision requires the creation of subgroups (i.e., relying on stratification) in order to study biomarkers (Gauld et al., 2020).

### 4.2. Clinical intuition is precision and stratification

These two methods of stratification and precision could precisely constitute the heart of the internal models of clinicians, i.e., of their clinical intuition. In terms of precision, clinicians' main goals are to be precise and detect the most specific behavioral elements (i.e., difference-makers) for a given patient (i.e., to increase *inter*-class variance). In this way, clinicians model each of the characteristics of his/her patients according to a set of data (information gathering), his/her theoretical background, the adaptation of his/her model and its dynamic corrections, as described in literature on clinical decision-making in medical education (Bhugra et al., 2011). He/she looks for the most precise and objective elements available, or, in other words, performs precision modeling. Through this effort of precision, clinical intuition is thus integrated into idiographic thinking, i.e., which considers the most relevant specificities of a given patient.

**Figure 1:**
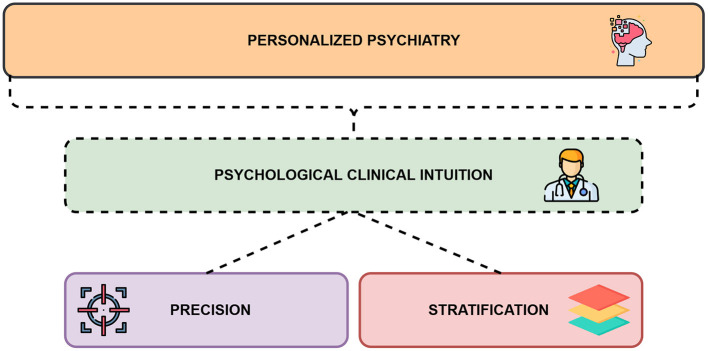
A framework for understanding personalized psychiatry and psychological clinical intuition together.

However, in parallel and regarding stratification, clinicians aim to consider the associated smallest homogeneous subgroup for which a diagnosis or a treatment is recognized (i.e., to decrease *intra*-class variance). They would rely on their diagnostic, predictive, prognostic and therapeutic heuristics, based on their capacity to conceptually understand a given patient based on perceived similarities in other patients previously encountered. This ability to generalize is offered by the possibility of stratifying patients. To stratify, clinicians utilize their previous experiences or their educational background (Meehl, 1967; Bhugra et al., 2011). In this way, clinical intuition is not only the result of individual, idiographic modeling, coming from the minds of clinicians, isolated from any theoretical support, reference group or nomothetic framework (i.e., sets of laws). As in the logic of stratification, which is based on subgroups of patients, clinicians base themselves on their own reference groups previously integrated into their internal model. The internal logic of policies and research programs based on stratification is therefore found in the clinician's nomothetic logic. Figure 1 shows the intricacies between personalized psychiatry and psychological clinical intuition, between precision and stratification.

## 5. Conclusion

To conclude, beyond its limits, personalized psychiatry could refine the process of modeling of a fuzzy clinical intuition in psychology. Such a modeling fits with two reasoning methods already intuitive for the clinician, aka the necessity to detect both the most precise elements of a given patient (precision) as well as define the finest subgroups to which he/she belongs (stratification). In this way, clinical intuition can be defined as a back-and-forth process between the precise targeting of data from clinical interviews and the clinician's use of subgroups of patients integrated during his/her learning and experience into his/her internal model. This explanation of clinical intuition, offered by the framework of personalized psychiatry, could be integrated into the already rich field of study of pedagogy in psychology, and enrich studies on decision-making.

In parallel, understanding personalized psychiatry as a challenge of precise and stratified clinical modeling could shed light on its practice in return. In addition to being useful for understanding clinical intuition in psychology, the interest of personalized psychiatry in rigorous and systematic modeling of clinical practice constitutes a truly original first step in the history of psychiatry and will certainly be an important future challenge for research and public health.

## Author contributions

CG: original draft preparation, writing, and conceptualization. YM: visualization, supervision, and final approval. PF: conceptualization, reviewing, and validation. All authors agree to be accountable for the content of the work. All authors contributed to the article and approved the submitted version.
